# A Mechanosensitive Channel Governs Lipid Flippase-Mediated Echinocandin Resistance in Cryptococcus neoformans

**DOI:** 10.1128/mBio.01952-19

**Published:** 2019-12-10

**Authors:** Chengjun Cao, Yina Wang, Seema Husain, Patricia Soteropoulos, Chaoyang Xue

**Affiliations:** a Public Health Research Institute, New Jersey Medical School, Rutgers University, Newark, New Jersey, USA; b The Genomics Center, New Jersey Medical School, Rutgers University, Newark, New Jersey, USA; c Department of Microbiology, Biochemistry and Molecular Genetics, New Jersey Medical School, Rutgers University, Newark, New Jersey, USA; University of Tennessee Health Science Center; University of Texas Health Science Center

**Keywords:** antifungal drug resistance, calcium signaling, *Cryptococcus neoformans*, fungi, lipid flippase

## Abstract

Echinocandins show fungicidal activity against common invasive mycoses but are ineffective against cryptococcosis. The underlying mechanism for echinocandin resistance in Cryptococcus neoformans remains poorly understood but has been shown to involve Cdc50, the regulatory subunit of lipid flippase. In a forward genetic screen for *cdc50*Δ suppressor mutations that are caspofungin resistant, we identified Crm1 (caspofungin resistant mutation 1), a homolog of mechanosensitive channel proteins, and showed that *crm1*Δ restored caspofungin resistance in *cdc50*Δ cells. Caspofungin-treated *cdc50*Δ cells exhibited abnormally high intracellular calcium levels ([Ca^2+^]c) and heightened activation of the calcineurin pathway. Deletion of *CRM1* in the *cdc50*Δ background normalized the abnormally high [Ca^2+^]c. Cdc50 interacts with Crm1 to maintain cellular calcium homeostasis. Analysis of chitin/chitosan content showed that deleting *CRM1* reversed the decreased chitosan production of *cdc50*Δ cells. Together, these results demonstrate that Cdc50 and Crm1 regulation of the calcineurin pathway and cytoplasmic calcium homeostasis may underlie caspofungin resistance in C. neoformans.

## Abstract (precis)

Cryptococcus neoformans is the leading cause of fungal meningitis, accounting for ∼15% of HIV/AIDS-related deaths, but treatment options for cryptococcosis are limited. Echinocandins are the newest fungicidal drug class introduced but are ineffective in treating cryptococcosis. Our previous study identified the lipid flippase subunit Cdc50 as a contributor to echinocandin resistance in C. neoformans. Here, we further elucidated the mechanism of Cdc50-mediated caspofungin drug resistance. We discovered that Cdc50 interacts with the mechanosensitive calcium channel protein Crm1 to regulate calcium homeostasis and caspofungin resistance via calcium/calcineurin signaling. These results provide novel insights into echinocandin resistance in this pathogen, which may lead to new treatment options, as well as inform echinocandin resistance mechanisms in other fungal organisms and, hence, advance our understanding of modes of antifungal drug susceptibility and resistance.

## INTRODUCTION

Invasive fungal infections are becoming more prevalent and problematic in immunocompromised populations, particularly those individuals with AIDS or on immunosuppressive therapies. Cryptococcus neoformans is a major human fungal pathogen and the causative agent of cryptococcal meningoencephalitis that accounts for ∼15% of AIDS-related deaths (1, 2). Fungal infections are solely treated by antifungal drugs that are very limited in options. The most common treatment options for cryptococcosis are either fungistatic (azoles) or toxic (amphotericin B) (3). Echinocandins are the newest approved antifungal drug class that targets β-1,3-glucan synthase, which synthesizes the key cell wall component β-1,3-glucan. However, this drug class is ineffective against *Cryptococcus* species (4–6), which is surprising because these organisms express the echinocandin target enzyme (7). Elucidating the mechanism of echinocandin resistance in C. neoformans should improve our understanding of these apparent contradictions and may enable the use of echinocandin drugs for treating cryptococcosis and other mycoses for which current drugs are ineffective.

In *Candida* and *Aspergillus* species, clinical resistance to echinocandins typically arises due to point mutations in the *FKS* genes (6, 8–14). For instance, in Candida albicans, the point mutations in *FKS1* reduce glucan synthase sensitivity to echinocandins, resulting in elevated MICs and reduced pharmacodynamic responses (8, 13). Mutations in *Candida FKS1* also change the expression levels of *FKS1* and chitin genes (14). C. neoformans *FKS1* is essential for viability, and the enzyme is sensitive to echinocandins *in vitro* (7); no *FKS1* point mutations have been identified in C. neoformans, and this organism shows innate resistance to echinocandins. Neither the *FKS1* expression level nor β-1,3-glucan synthase localization changed after caspofungin treatment (15). These studies suggest that C. neoformans possesses an unidentified echinocandin resistance mechanism, allowing the cells to survive in the presence of echinocandins.

Some evidence suggests that cells can tolerate echinocandin exposure by upregulating compensatory cell wall salvage mechanisms (16–19). For instance, treatment with echinocandins, such as caspofungin, inhibits the synthesis of cell wall β-1,3-glucan and leads to a compensatory increase in cell wall chitin synthesis, helping to restore cell wall integrity (16, 18). Several pathways have been implicated in regulating echinocandin tolerance, in particular, the calcium (Ca^2+^)-sensitive calcineurin signaling pathway, which has been proposed to control the glucan-chitin interaction through the transcriptional regulation of chitin synthases (20, 21). Thus, it is possible that these pathways contribute to innate echinocandin resistance in C. neoformans. However, their involvement in this process has not yet been investigated extensively.

Recently, Pianalto et al. performed a forward genetic screen to identify cellular processes that mediate the relative tolerance to caspofungin and found several pathways contributing to caspofungin resistance in C. neoformans (15). Mutants of the calcineurin A catalytic and B regulatory subunits (*cna1*Δ or *cnb1*Δ) were hypersensitive to caspofungin (15, 22). Calcineurin is a known multifunctional regulator in fungi that influences fungal virulence and stress responses (23, 24). It has been reported that fluconazole had a synergistic effect with the calcineurin inhibitors FK506 and cyclosporine (CsA) in C. albicans (25, 26). Calcineurin signaling also regulates echinocandin resistance in fungal pathogens, such as *Candida* species and Aspergillus fumigatus (23, 27–29). A synergistic drug effect between the calcineurin inhibitor FK506 and caspofungin has also been reported in C. neoformans (24). Crz1 is a known downstream transcription factor of the calcineurin pathway in C. neoformans (30). However, recent study demonstrated that calcineurin likely has additional downstream effectors besides Crz1 in response to caspofungin treatment (15).

Our previous work showed that deletion of *CDC50*, which encodes the β-subunit of membrane lipid translocase (flippase), sensitizes C. neoformans to caspofungin and another glucan synthase inhibitor, MK-3118 (31). In addition to contributing to caspofungin resistance, Cdc50 is also essential for C. neoformans virulence in murine infection models (31). Lipid flippase mediates translocation of certain phospholipids across the plasma membrane to maintain the asymmetric distribution of phospholipids in the lipid bilayer membrane (32). How lipid flippase function mediates echinocandin drug resistance and fungal virulence remains unclear.

In this study, we performed a forward genetic screen to identify caspofungin-resistant *cdc50*Δ suppressor mutations to elucidate the mechanisms underlying Cdc50-mediated caspofungin resistance. We identified a putative mechanosensitive (MS) calcium channel protein, Crm1, whose mutation led to high drug resistance of the *cdc50*Δ mutant. Our data indicate that Cdc50 interacts with Crm1 to control intracellular calcium levels ([Ca^2+^]c) and thus governs caspofungin resistance. These results demonstrate that Crm1 is required for Cdc50-mediated echinocandin drug resistance in C. neoformans.

## RESULTS

### Screen for mutations conferring caspofungin resistance to *cdc50*Δ mutants.

Our previous study identified Cdc50 as required for caspofungin resistance in C. neoformans, and the *cdc50*Δ null mutant is hypersensitive to caspofungin (31). To elucidate the underlying mechanism of Cdc50-mediated drug resistance, we screened for caspofungin-resistant *cdc50*Δ mutants by inoculating the *cdc50*Δ mutant into medium supplemented with a high concentration of caspofungin and selecting spontaneous mutant suppressors that grow under this condition (see Fig. S1A in the supplemental material). Two *cdc50*Δ mutants (mutant 1 and mutant 2, named M1 and M2, respectively) showed stable resistance to caspofungin following multiple passages on drug-free medium. To characterize caspofungin sensitivity in M1 and M2, we used an agar-based spot assay to test the comparative growth of these two mutant strains on yeast extract-peptone-dextrose (YPD) medium supplemented with different concentrations of caspofungin (Fig. S1B). There were no growth defects in either strain on YPD medium without drug. Both M1 and M2 displayed growth rates comparable to the growth of H99 and much better growth than the original *cdc50*Δ mutant on YPD medium containing 32 μg/ml or less caspofungin. However, both suppressor mutants are less resistant to 64 μg/ml caspofungin than the wild type. Overall, our data showed that M1 and M2 reversed caspofungin sensitivity of the *cdc50*Δ mutant.

10.1128/mBio.01952-19.1 FIG S1 Isolating caspofungin resistance in *cdc50*Δ mutant. (a) Scheme of screen strategy for caspofungin resistant *cdc50*Δ mutants. (b) Cultures of strains grown overnight in YPD medium and diluted to an *A*_600_ of 1.0. Tenfold serial dilutions were prepared, and 5 μl of each suspension was spotted on YPD agar supplemented with 0, 16, 32, or 64 μg/ml caspofungin (CAS). M1, mutant 1; M2, mutant 2. Download FIG S1, TIF file, 2.2 MB. Copyright © 2019 Cao et al. 2019 Cao et al. This content is distributed under the terms of the Creative Commons Attribution 4.0 International license.

### Analysis of HS regions of *FKS1* genes in M1 and M2.

Amino acid substitutions associated with resistance occur in two limited but highly conserved hot spot (HS) regions of the β-1,3-glucan synthase protein sequences in *Candida* and *Aspergillus* species (8–13). In C. neoformans, there is a single *FKS1* homolog, which contains two conserved HS regions (33). Therefore, we tested whether M1 and M2 isolates contain mutations in the HS regions of *FKS1*. Sequences of the HS1 or HS2 region in M1 and M2 were compared to the corresponding sequence of the original *cdc50*Δ null mutant or wild-type H99. We did not find any mutations in the HS regions of *FKS1* (data not shown). These data suggest that caspofungin resistance of M1 and M2 mutants is independent of HS region mutations in the *FKS1* gene.

### Genomic sequence analysis of M1 and M2.

To identify DNA changes in the mutants, we performed whole-genome resequencing (Mid-Seq) to analyze the two caspofungin-resistant *cdc50*Δ mutants. By comparing M1 and M2 genome sequence data with the H99 genomic sequence, we identified all variants, including base deletions, insertions, inversions, and translocations, in the whole genome (Table S4). We found 215 variants in the M1 genome and 198 variants in the M2 genome. In total, we identified eight proteins that had amino acid changes in either M1 or M2 (Table S1).

10.1128/mBio.01952-19.3 TABLE S1 Genes containing mutations in M1 and M2. Download Table S1, DOCX file, 0.01 MB. Copyright © 2019 Cao et al. 2019 Cao et al. This content is distributed under the terms of the Creative Commons Attribution 4.0 International license.

10.1128/mBio.01952-19.4 TABLE S2 MICs of caspofungin in YPD supplemented with calcium chelator BAPTA. Download Table S2, DOCX file, 0.01 MB. Copyright © 2019 Cao et al. 2019 Cao et al. This content is distributed under the terms of the Creative Commons Attribution 4.0 International license.

10.1128/mBio.01952-19.5 TABLE S3 Primers used in this study. Download Table S3, DOCX file, 0.01 MB. Copyright © 2019 Cao et al. 2019 Cao et al. This content is distributed under the terms of the Creative Commons Attribution 4.0 International license.

10.1128/mBio.01952-19.6 TABLE S4 Complete list of mutation sites identified by genome comparison. Download Table S4, XLSX file, 0.2 MB. Copyright © 2019 Cao et al. 2019 Cao et al. This content is distributed under the terms of the Creative Commons Attribution 4.0 International license.

To determine if any of these genes contributed to caspofungin resistance in C. neoformans, we tested the caspofungin susceptibility of both null and double mutants between the *cdc50*Δ strain and each single mutant. The caspofungin sensitivities of all null mutants were similar to those of wild-type strain H99 using agar plates and MIC assays (data not shown). Agar-based spot assays indicated that one double mutant between the *crm1*Δ strain (caspofungin resistance mutant 1, deletion of CNAG_01704) and the *cdc50*Δ strain (*crm1*Δ *cdc50*Δ mutant) was resistant to caspofungin at a level similar to the levels of the M1 and M2 mutants. Other double mutants showed caspofungin sensitivity similar to that of the original *cdc50*Δ mutant (data not shown). These results demonstrate that Crm1 participates in Cdc50-mediated caspofungin resistance in C. neoformans.

### Disrupting *CRM1* rescues caspofungin resistance in the *cdc50*Δ mutant.

**FIG 1:**
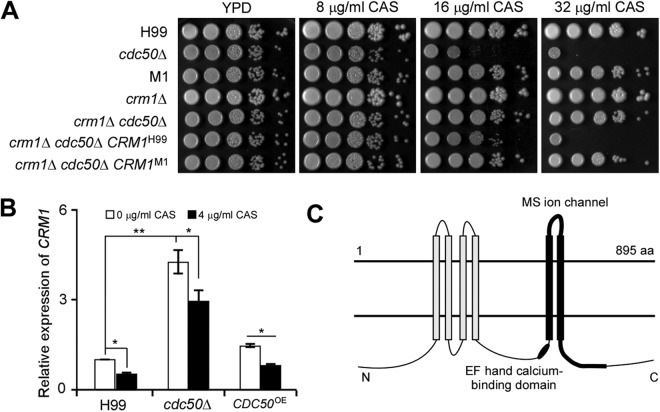
Deleting *CRM1* rescues caspofungin sensitivity in the *cdc50*Δ mutant. (A) An agar-based spotting assay measured caspofungin (CAS) sensitivity. Cultures were grown overnight in YPD medium and adjusted to a starting concentration at an *A*_600_ of 1.0. Tenfold serial dilutions were prepared, and 5 μl of each suspension was spotted on YPD agar supplemented with 0, 16, or 32 μg/ml caspofungin. Prior to being photographed, plates were incubated for 4 days at 30°C. (B) Relative expression levels of *CRM1* in the wild type, *cdc50*Δ mutant, and *CDC50* overexpression strain. Yeast cells collected from overnight culture in YPD medium were replated onto YPD medium containing 0 or 4 μg/ml of caspofungin. Cells were incubated for an additional 16 h at 30°C before RNA extraction for quantitative RT-PCR analysis. The *GAPDH* gene served as a reference. The expression level of *CRM1* under the YPD condition was set as 1. The data shown are cumulated from three independent experiments. Statistical analysis was done by a two-tailed *t* test. *, *P* < 0.05; **, *P* < 0.01. (C) Predicted Crm1 membrane topology. White rods represent transmembrane domains. The black rod represents a predicted EF-hand Ca^2+^-binding motif. The thick black line indicates the predicted mechanosensitive (MS) channel. aa, amino acids.

The *CRM1* gene in the M1 genome contains two nucleotide deletions at positions 343 and 344 that produce a stop codon (Table S1), prematurely terminating Crm1 protein. To confirm the role of Crm1 in caspofungin resistance in C. neoformans, we introduced the *CRM1* allele from M1 into the *crm1*Δ *cdc50*Δ double mutant. The genomic fragments containing the *CRM1* open reading frame (ORF) and its promoter were amplified from M1 and the wild-type strain H99 (as a control). Each *CRM1* fragment was introduced into the *crm1*Δ *cdc50*Δ double mutant to generate two *CRM1* complement strains (*crm1*Δ *cdc50*Δ *CRM1*^H99^ or *crm1*Δ *cdc50*Δ *CRM1*^M1^ strain). The *crm1*Δ *cdc50*Δ *CRM1*^H99^ strain showed increased caspofungin sensitivity, while the *crm1*Δ *cdc50*Δ *CRM1*^M1^ strain showed drug resistance similar to that of the *crm1*Δ *cdc50*Δ double mutant. These results suggest the *CRM1* gene in M1 is nonfunctional (Fig. 1A). Furthermore, *CRM1* expression levels were significantly induced in the *cdc50*Δ mutant compared to levels in the wild type and decreased in all strains following caspofungin treatment (Fig. 1B). Based on these results, we conclude that Crm1 negatively regulates caspofungin sensitivity of the *cdc50*Δ mutant and drives the increased caspofungin resistance in the M1 mutant.

Cdc50 is required to develop fungal virulence factors, including growth at 37°C, production of melanin and capsule, cellular integrity, and stress resistance (31). Therefore, we examined the development of virulence factors and stress responses in the *crm1*Δ mutant and the *crm1*Δ *cdc50*Δ double mutant. Under the tested stress conditions, the *crm1*Δ mutant showed a phenotype similar to that of wild-type H99, while the phenotype of the *crm1*Δ *cdc50*Δ double mutant resembled that of the *cdc50*Δ mutant (Fig. S2A). We also investigated a possible role for Crm1 in fungus-macrophage interactions by measuring the phagocytosis rate and intracellular proliferation of these strains in the J774 macrophage cell line. We did not observe any obvious differences between the *crm1*Δ mutant and H99 or between the *cdc50*Δ mutant and the *crm1*Δ *cdc50*Δ double mutant (Fig. S2B to D). Taken together, these data suggest that Crm1 mediates caspofungin resistance but not other Cdc50-dependent functions under these conditions.

10.1128/mBio.01952-19.2 FIG S2 Crm1 is not involved in the resistance of stress responses, classical virulence factor development, and survival in macrophages. (A) Cultures of H99, *cdc50Δ*, M1, *crm1*Δ, *crm1*Δ *cdc50*Δ, *crm1*Δ *cdc50*Δ *CRM1*^H99^, and *crm1*Δ *cdc50*Δ *CRM1*^M1^ strains were prepared with serial dilutions and were grown on YPD medium containing 0.03% SDS, 1 M KCl, 250 μg/ml calcofluor white (CFW), and 0.5% Congo red. Cells were also grown on l-DOPA medium. The results were photographed after 4 days of incubation. Their growth on YPD medium at 37°C was determined by serial dilutions. (B) The phagocytosis rate of C. neoformans in the J774 macrophages was measured in 96-well plates. PBS-washed C. neoformans H99, *crm1*Δ, *cdc50Δ*, M1, and *crm1*Δ *cdc50*Δ strains were added to the activated macrophages and incubated for 2 h at 37°C with 5% CO_2_. The percentage of macrophages containing internalized yeast cells was measured from over 100 macrophages following Giemsa staining. (C) The number of yeast cells in each macrophage was also calculated under an inverted microscope. Data were generated from counting over 500 macrophages. (D) Intracellular proliferation of C. neoformans in the macrophage cell line. Fungal strains were added to the activated macrophages and incubated for 2 hours at 37°C with 5% CO_2_. Nonadherent extracellular yeast cells were then removed by washing with fresh DMEM. Numbers of fungal CFU from macrophage cultures after an additional 0, 4, or 24 hours of incubation were used to determine intracellular proliferation and macrophage killing. Error bars indicate standard deviations for three experiments. Download FIG S2, TIF file, 2.8 MB. Copyright © 2019 Cao et al. 2019 Cao et al. This content is distributed under the terms of the Creative Commons Attribution 4.0 International license.

### Calcium positively regulates caspofungin resistance.

Based on protein structure prediction using the HMMTOP, version 2.0, program, we revealed that Crm1 shares sequence similarity with the MscS-like (mechanosensitive channels of small conductance-like, or MSL) protein family, members of which resemble bacterial MscS in their transmembrane segment and vicinity (34). Crm1 is predicted to have six transmembrane (TM) domains, with TM5 and TM6 helixes predicted to form a mechanosensitive channel. Crm1 also contains a conserved EF-hand calcium-binding domain (Fig. 1C), which could be important for calcium channel function. Because we identified Crm1 as a potential member of the EF-MSL family, we propose that calcium signaling and [Ca^2+^]c may regulate caspofungin resistance in C. neoformans.

**FIG 2:**
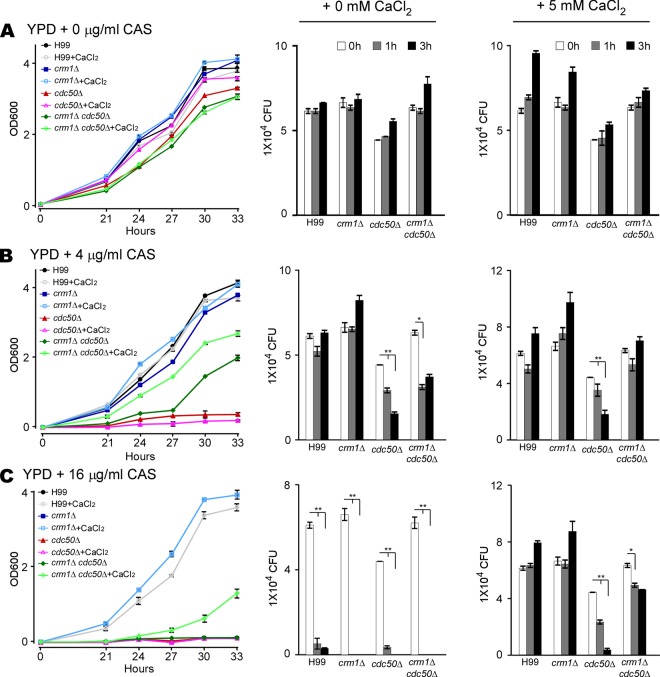
Calcium levels influence cell growth and survival rate of C. neoformans. (A to C) Growth curves (left) and survival rates in YPD medium (middle) or YPD medium with 5 mM CaCl_2_ (right). Cultures of H99, *cdc50*Δ, *crm1*Δ, and *crm1*Δ *cdc50*Δ strains were grown on YPD medium containing 0, 4, or 16 μg/ml of caspofungin (CAS), as indicated, and incubated for 33 h at 30°C. Cell density was determined by measuring the optical density at 600 nm (OD_600_) at different time points, as indicated. The number of yeast CFU/ml was determined at different time points after incubation by plating samples onto drug-free medium. Triplicates were used for each measurement. *, *P* < 0.05; **, *P* < 0.01 (two-tailed *t* test).

To determine the potential effect of calcium levels on caspofungin sensitivity, we measured the MIC of caspofungin in liquid YPD medium supplemented with different concentrations of calcium chloride (CaCl_2_) or a calcium chelator (EGTA) (Table 1). We found no differences in MICs between the *cdc50*Δ mutant and the *crm1*Δ *cdc50*Δ double mutant on YPD medium without CaCl_2_ supplement. However, the *crm1*Δ *cdc50*Δ mutant had a higher MIC than the *cdc50*Δ mutant with CaCl_2_ addition. To further understand the link between Ca^2+^ and caspofungin resistance, we measured fungal growth rates in liquid YPD medium with different concentrations of caspofungin, with or without 5 mM CaCl_2_ (Fig. 2). Supplementation with 5 mM CaCl_2_ in YPD medium did not affect cell growth of any strain without caspofungin treatment (Fig. 2A). Both the *cdc50*Δ mutant and the *crm1*Δ *cdc50*Δ double mutant showed growth defects in the presence of caspofungin without exogenous CaCl_2_. Supplementation with exogenous CaCl_2_ rescued the growth defects in the wild-type, *crm1*Δ, and *crm1*Δ *cdc50*Δ strains but not in the *cdc50*Δ mutant (Fig. 2B and C). Together, these data demonstrate that calcium increases C. neoformans resistance to caspofungin killing.

**TABLE 1:** MICs of caspofungin in YPD medium supplemented with additional calcium or a calcium chelator (EGTA)

Strain	MIC (μg/ml) in YPD medium with:								
	EGTA (μg/ml)					CaCl_2_ (mM)			
	2	1	0.5	0.25	0	2.5	5	10	20
H99	8	8	16	16	16	32	64	64	128
*cdc50*Δ strain	2	2	4	4	4	4	8	8	8
*crm1*Δ strain	8	8	8	8	8	32	64	64	128
*crm1*Δ *cdc50*Δ strain	2	2	2	4	4	16	32	32	64
*CRM1*^OE^ strain	16	16	16	16	16	16	32	32	128

### The calcineurin pathway drives Cdc50-mediated caspofungin resistance in C. neoformans.

**FIG 3:**
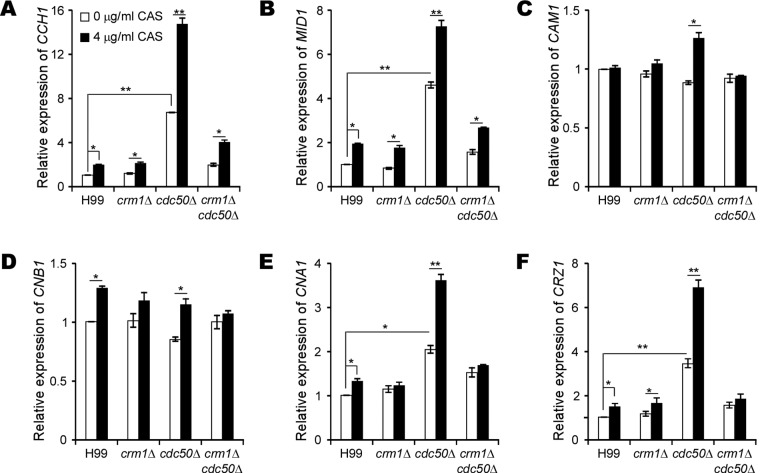
Crm1 is required to activate the calcineurin pathway. (A to F) Relative expression levels of *CCH1* , *MID1*, *CAM1* , *CNB1*, *CNA1*, and *CRZ1*, as indicated, were determined. Cultures of H99, *crm1*Δ, and *crm1*Δ *cdc50*Δ strains were incubated overnight in YPD medium and replated onto YPD medium containing 0 or 4 μg/ml caspofungin (CAS). Cultures were incubated for 16 h at 30°C before RNA extraction for quantitative RT-PCR analysis. The data shown are cumulated from three independent experiments. The *GAPDH* gene served as a reference. The expression levels of each gene under the YPD-only condition was set as 1. *, *P* < 0.05; **, *P* < 0.01 (two-tailed *t* test).

To delineate the potential role of the calcineurin pathway in caspofungin resistance in C. neoformans, we performed quantitative reverse transcription-PCR (RT-PCR) to measure the expression of genes involved in calcium signaling, including those encoding membrane calcium channel proteins *CCH1*, *MID1*, Ca^2+^ activated-calmodulin (*CAM1*), the catalytic and regulatory subunits of calcineurin (*CNA1* and *CNB1*), and the calcineurin-dependent transcription factor (*CRZ1*). Indeed, we found that caspofungin treatment upregulated these genes, especially in the *cdc50*Δ mutant, suggesting activation of the calcineurin pathway (Fig. 3). Such high induction of gene expression in the *cdc50*Δ mutant by caspofungin was reversed by deleting *CRM1*, suggesting that the calcineurin pathway was induced by caspofungin in the wild type and was hyperactivated in the *cdc50*Δ mutant in a Crm1-dependent manner.

To further confirm the function of calcineurin in Cdc50-mediated caspofungin resistance in C. neoformans, we examined the MIC of caspofungin in the presence of the calcineurin inhibitor, cyclosporine (CsA) (Table 2). The *cdc50*Δ mutant showed a stronger tolerance to caspofungin than the *crm1*Δ *cdc50*Δ double mutant in the presence of CsA. Intriguingly, both the M1 mutant and *crm1*Δ *cdc50*Δ double mutant exhibited high CsA sensitivity in comparison to that of H99 or the *crm1*Δ strain. These data indicate that the calcineurin pathway-mediated caspofungin resistance is dependent on Cdc50 and Crm1.

**TABLE 2:** Synergistic activity of caspofungin combined with CsA at 37°C

Strain	CsA MIC (μg/ml)	Caspofungin MIC (μg/ml) in YPD with CsA at the indicated concn (μg/ml)^*a*^						
		0	0.0625	0.125	0.25	0.5	1	2
H99	4	32	32	32	32	16	8	2
*crm1*Δ strain	4	32	32	32	32	16	8	2
M1	0.25	16	1	≤0.5	NA	NA	NA	NA
*cdc50*Δ strain	1	8	8	8	4	1	NA	NA
*crm1*Δ *cdc50*Δ strain	0.25	16	1	≤0.5	NA	NA	NA	NA

a NA, not available.

### Disrupting Crm1 restores intracellular calcium homeostasis and reduces phosphatidylserine (PS) exposure and reactive oxygen species (ROS) generation in the *cdc50*Δ mutant following caspofungin treatment.

**FIG 4:**
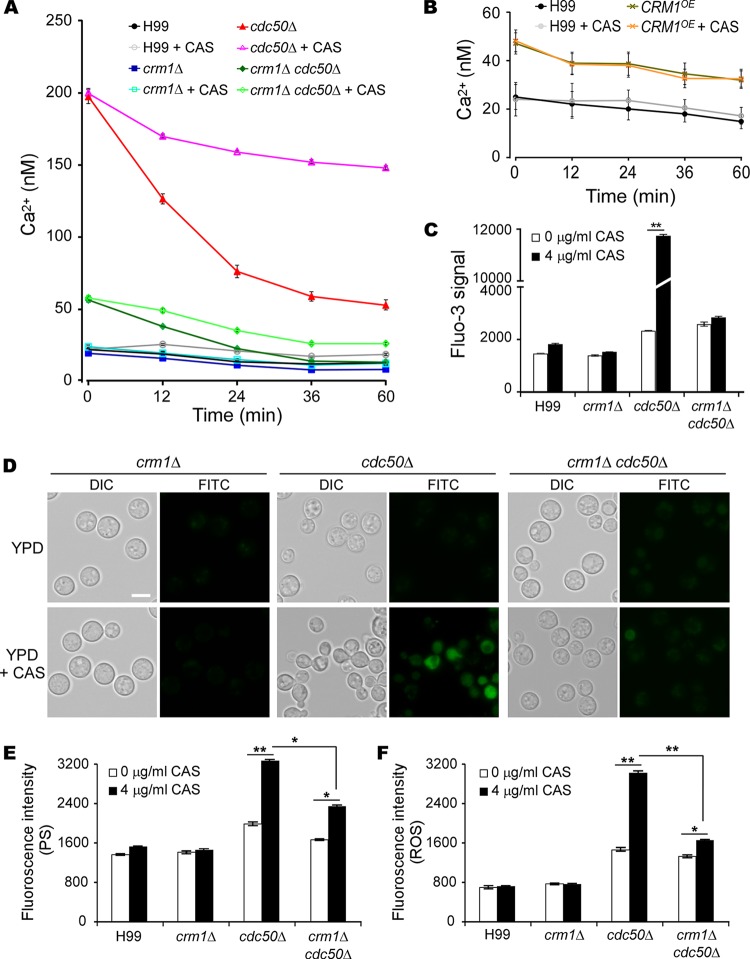
Loss of Crm1 restores calcium homeostasis and decreases cell surface PS exposure and ROS production in the *cdc50*Δ mutant. Intracellular calcium levels, PS signal, and ROS signal were determined at the indicated time points by flow cytometry. Overnight cultures of the H99, *cdc50*Δ, *crm1*Δ, *crm1*Δ *cdc50*Δ and *CRM1*^OE^ strains were resuspended in YPD containing 0 or 4 μg/ml caspofungin. (A) Time-lapse measurement of intracellular calcium levels in the presence and absence of caspofungin (CAS). (B) Intracellular calcium levels in the *CRM1*^OE^ strain. (C) Fluorescent signal intensities of intracellular calcium levels after 16 h of incubation in the presence or absence of caspofungin treatment. (D) The fluorescent signal of fungal cells was detected by fluorescence microscopy after annexin V binding. Bar, 10 μm. (E and F) Quantification of fluorescent signal intensities of cell surface PS and ROS, as indicated, using flow cytometry for 100,000 yeast cells. Triplicates were used for each measurement. *, *P* < 0.05; **, *P* < 0.01 (two-tailed *t* test).

Given the EF-hand motif in Crm1 and a clear role of calcium levels in caspofungin resistance in C. neoformans, we monitored the dynamic changes of [Ca^2+^]c during caspofungin treatment. Cytosolic Ca^2+^ levels in the wild-type, *crm1*Δ, *cdc50*Δ, *crm1*Δ *cdc50*Δ, and *CRM1*-overexpressing (*CRM1*^OE^) strains were measured in a flow cytometry assay with Fluo-3 acetoxymethyl ester (Fluo-3/AM) indicator (Fig. 4A to C). We found that the *cdc50*Δ mutant had a much higher [Ca^2+^]c than the wild type and other mutants (Fig. 4A). The cytosolic Ca^2+^ concentration in the *cdc50*Δ mutant decreased rapidly without drug treatment but was maintained at a high level in the presence of caspofungin. Disrupting *CRM1* in the *cdc50*Δ mutant resulted in decreased Ca^2+^ levels, especially in the absence of caspofungin, compared to the level in the *cdc50*Δ mutant. Both a higher [Ca^2+^]c in the *CRM1*^OE^ strain than in the wild type (Fig. 4B) (*P* < 0.05 by two-tail *t* test) and an increased *CRM1* expression level in the *cdc50*Δ mutant (Fig. 1B) suggest that Crm1 is involved in Cdc50-regulated calcium homeostasis in C. neoformans. In addition, we detected a significant increase in Ca^2+^ concentration in *cdc50*Δ cells but not in other strains after a longer incubation with caspofungin (16 h) (Fig. 4C). These results indicate that caspofungin treatment significantly induces and maintains high [Ca^2+^]c in the *cdc50*Δ mutant and that loss of *CRM1* restores calcium homeostasis in the *cdc50*Δ mutant.

Excessive [Ca^2+^]c may promote cell death (35), which is often associated with increased phosphatidylserine (PS) exposure on the cell surface and a burst of reactive oxygen species (ROS) (36). We sought to test our hypothesis that high Ca^2+^ levels in the *cdc50*Δ mutant under caspofungin stress facilitate cell death. Therefore, we measured cell surface PS levels using fluorescein isothiocyanate (FITC)-conjugated annexin V staining (FITC-annexin) and monitored ROS generation with the dye indicator dichlorodihydrofluorescein diacetate (H_2_DCFDA) (Fig. 4D to F). The *cdc50*Δ mutant cells treated with caspofungin exhibited significantly elevated FITC and ROS levels. Although the *crm1*Δ *cdc50*Δ double mutant also showed increased ROS generation and PS exposure with caspofungin treatment, their signal intensity was much lower than levels in the *cdc50*Δ mutant. No change in fluorescence signal was detected either in the wild type or *crm1*Δ mutant. Based on these results, we conclude that caspofungin treatment induces cell death in the *cdc50*Δ mutant, as indicated by increased cell surface PS exposure and high ROS production.

### Cdc50 interacts with Crm1 to regulate caspofungin uptake in C. neoformans.

**FIG 5:**
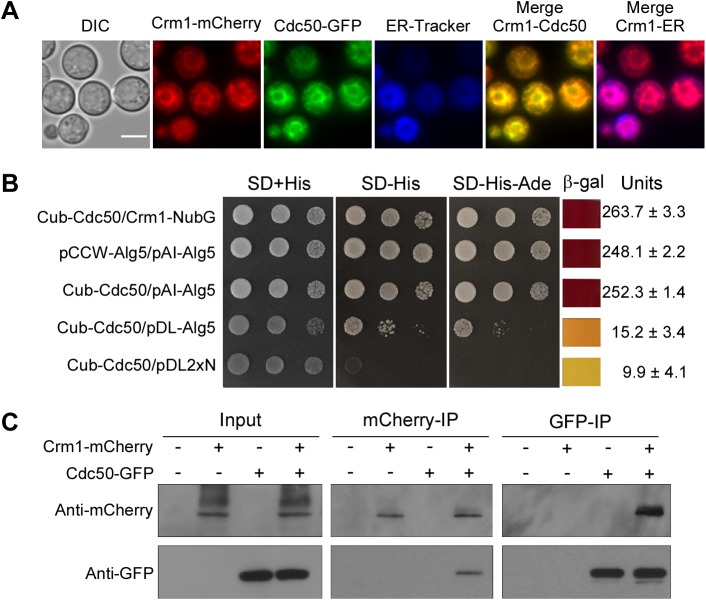
Cdc50 interacts with Crm1. (A) Colocalization of Crm1 and Cdc50. Fluorescent signals generated by Crm1-mCherry and Cdc50-GFP in cells grown in YPD medium. DIC, differential interference contrast. Bar, 5 μm. (B) Interactions between Crm1 and Cdc50 were observed in the split-ubiquitin system. The C-terminal half of ubiquitin (Cub) was fused to the N terminus of Cdc50 cDNA (Cub-Cdc50). The N-terminal half of ubiquitin (NubG) was fused to the N terminus of Crm1 cDNA (NubG-Crm1). Yeast transformants were grown on selective medium. β-Galactosidase activity assays were performed to verify the interaction. Values were averaged from two independent experiments. SD, synthetic dextrose. (C) Total proteins from cells expressing Crm1-mCherry, Cdc50-GFP, or both Crm1-mCherry and Cdc50-GFP were extracted. The potential Crm1-Cdc50 interaction was analyzed by coimmunoprecipitation (IP) with anti-mCherry or anti-GFP antibody and evaluated by immunoblotting.

The *cdc50*Δ mutant showed a significant increase in caspofungin uptake, which likely contributes to its hypersensitivity to caspofungin (31). To determine how Crm1 promotes caspofungin drug susceptibility in the *cdc50*Δ mutant, we first analyzed Crm1 and Cdc50 colocalization by expressing a Crm1-mCherry fusion protein in the Cdc50-green fluorescent protein (GFP) background. We observed that the localization of Crm1 on the endoplasmic reticulum (ER) membrane overlapped that of Cdc50 (Fig. 5A).

Because Cdc50 and Crm1 colocalized on the ER membrane and because the *CRM1* gene was overexpressed in the *cdc50*Δ mutant, we tested the hypothesis that Cdc50 interacts with Crm1 to control its expression to maintain intracellular calcium homeostasis and regulate caspofungin resistance. Indeed, we detected a direct interaction between Crm1 and Cdc50 in a membrane-based yeast two-hybrid protein-protein interaction system (split ubiquitin system) that we have employed in previous studies (37, 38). We generated a fusion plasmid constructed by fusing the C-terminal half of ubiquitin (Cub) to the N terminus of full-length Cdc50 cDNA (Cub-Cdc50). The Crm1 construct was generated by fusing full-length Crm1 with the mutated N-terminal half of ubiquitin (NubG), in which Ile-13 is replaced by Gly. Transformants coexpressing Cub-Cdc50 and NubG-Crm1 grew on medium lacking histidine and adenine and produced robust β-galactosidase enzyme activity, indicating a direct interaction between Cdc50 and Crm1 (Fig. 5B). This interaction was confirmed by membrane protein coimmunoprecipitation. The total protein from the strain expressing both Crm1-mCherry and Cdc50-GFP was purified, and protein complex was immunoprecipitated with anti-mCherry or anti-GFP antibodies. Western blotting results further demonstrated that Crm1 interacts with Cdc50 (Fig. 5C).

**FIG 6:**
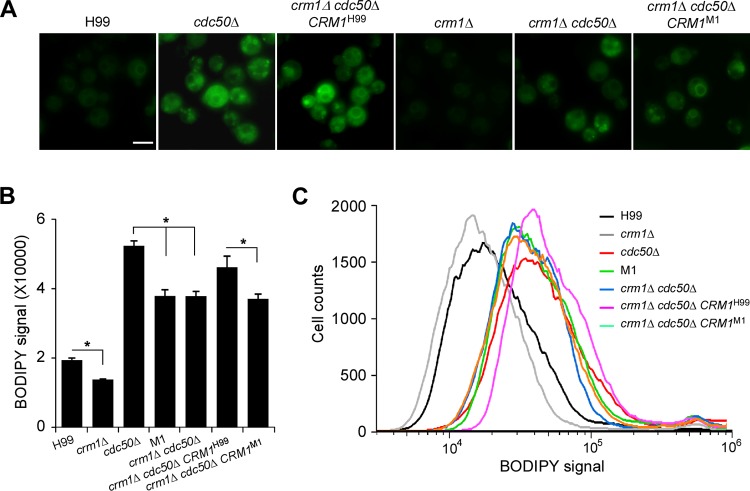
Regulation of Crm1 in caspofungin uptake in C. neoformans. (A) Fungal cultures were coincubated with 5 μM BODIPY-labeled caspofungin for 30 min at 30°C. The fluorescent signal of fungal cells was detected by fluorescence microscopy. (B) Fluorescent signal intensity was quantified using flow cytometry for 100,000 yeast cells treated with 5 μM BODIPY-caspofungin. Triplicates were used for each measurement. *, *P* < 0.05. (C) Representative images of fluorescent signal quantification results using flow cytometry.

The caspofungin uptake ability of the wild type and mutants was measured using boron dipyrromethene difluoride (BODIPY)-labeled caspofungin (39) (Fig. 6A). Mutants containing a *CDC50* deletion (*cdc50*Δ and *crm1*Δ *cdc50*Δ strains) had higher levels of fluorescent signal than the H99 and *crm1*Δ strains. Quantifying the fluorescent signal intensity showed that disrupting *CRM1* reduced BODIPY-caspofungin uptake in the *cdc50*Δ mutant (Fig. 6B and C). Taken together, these data suggest that Crm1 regulates caspofungin resistance at least in part through altering drug uptake ability.

### Caspofungin treatment induces a compensatory increase in chitin/chitosan content in *cdc50*Δ cells.

**FIG 7:**
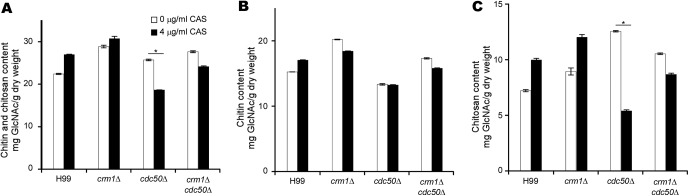
Cdc50 and Crm1 contribute to the regulation of chitin and chitosan production in C. neoformans. (A to C) Chitin and chitosan production of the H99, *crm1*Δ, *cdc50*Δ, and *crm1*Δ *cdc50*Δ strains in the presence or absence of caspofungin (CAS). Cells were grown on YPD medium overnight as described in Materials and Methods. *, *P* < 0.01.

Caspofungin treatment induces a marked increase in chitin synthesis in C. albicans and A. fumigatus (16, 18). The cryptococcal cell wall also contains chitin and significant amounts of chitosan, the deacetylated form of chitin, which is produced by chitin deacetylase enzymes that remove acetyl groups from nascent chitin polymers (40, 41). Strains of C. neoformans with reduced chitosan levels are more sensitive to diverse cell wall stresses (42). To understand the potential change of chitin and chitosan following caspofungin treatment in C. neoformans, changes in cell wall chitin/chitosan content in wild-type and mutant strains treated with caspofungin were investigated (Fig. 7). Quantification of individual cell wall chitin and chitosan levels was performed as described by Baker et al. (43). Our data showed that in the presence of caspofungin, the production of chitin/chitosan increased in the H99 and *crm1*Δ strains but significantly decreased in the *cdc50*Δ mutant. Although the *crm1*Δ *cdc50*Δ double mutant treated with caspofungin showed decreased chitin/chitosan content, it also produced more than the *cdc50*Δ mutant (Fig. 7A). We found there were 1.4- and 1.3-fold increases in the average chitosan levels in the H99 and *crm1*Δ strains following caspofungin treatment, respectively, while the *cdc50*Δ mutant treated with caspofungin showed a 2.3-fold decrease in cell wall chitosan compared to the level of the untreated controls (Fig. 7C). The chitosan content of the *crm1*Δ *cdc50*Δ mutant was significantly higher than that of the *cdc50*Δ mutant, with a 1.1-fold decrease in the presence of caspofungin. Taken together, these results indicate that treatment with caspofungin significantly decreased chitosan content in *cdc50*Δ cells and that deletion of *CRM1* rescued its reduced chitosan level, suggesting that the chitosan level is important for caspofungin resistance in C. neoformans.

## DISCUSSION

In this study, we used an unbiased mutagenesis approach to search for suppressor mutations of *cdc50*Δ that can rescue its caspofungin-sensitive phenotype and thereby identified the mechanosensitive calcium channel protein, Crm1. Loss of Crm1 function in the *cdc50*Δ mutant reversed its caspofungin sensitivity without changing other Cdc50-dependent functions. These data suggest that Crm1 is a specific regulator of Cdc50-dependent caspofungin resistance in C. neoformans. Crm1 directly interacts with Cdc50, and its expression was negatively regulated by Cdc50 and by caspofungin treatment. Caspofungin treatment in the *cdc50*Δ mutant significantly increased [Ca^2+^]c in the mutant, which may underline the observed rapid cell death and explain its hypersensitivity to caspofungin. Deleting the *CRM1* gene normalized this abnormally high [Ca^2+^]c, and as a result the double mutant regained caspofungin resistance. However, although suppressor mutants M1 and M2 restored the caspofungin resistance in the *cdc50*Δ mutant when the drug concentration was low, they were still more sensitive to a high drug concentration (64 μg/ml) than the wild type. Other proteins involved in caspofungin resistance have been recently reported (15). Therefore, it is possible that there are other parallel mechanisms controlling caspofungin resistance besides Crm1 in C. neoformans. This possibility will be studied in the future.

Crm1 is an EF-hand MscS-like protein (EF-MSL protein), characterized by one EF-hand Ca^2+^-binding domain upstream of the conserved mechanosensitive channel and several transmembrane domains (44, 45). This protein family is better described in bacteria, yet its function in fungi remains poorly understood. There is no sequence homolog of MscS-like proteins in Saccharomyces cerevisiae. The only reported study of fungal MscS-like proteins was performed in Schizosaccharomyces pombe, in which two MscS-like proteins, Msy1 and Msy2, were found to constitute two mechanosensitive channels that control [Ca^2+^]c and cell volume following hypo-osmotic shock (46, 47). Upon hypo-osmotic shock, the *msy1*Δ *msy2*Δ mutant displayed greater cell swelling than wild-type cells before undergoing cell death, which was enhanced by the influx of extracellular calcium, leading to an abnormally high [Ca^2+^]c in this double mutant. Interestingly, Msy1 and Msy2 appear to have distinct roles in maintaining cellular calcium homeostasis in S. pombe because they have different ER membrane localizations, and [Ca^2+^]c was increased in the *msy1*Δ mutant and decreased in the *msy2*Δ mutant (46). In C. neoformans, we found that Crm1 as the single MSL protein homolog contributed to caspofungin resistance, which suggests the potential involvement of the Ca^2+^ signaling pathway and intracellular Ca^2+^ homeostasis in conferring echinocandin drug resistance. However, our analysis did not reveal a clear function of Crm1 in osmotic regulation or cell swelling.

Previous publications demonstrated a link between Ca^2+^ signaling pathways and caspofungin resistance in *Candida* species and Aspergillus fumigatus (20, 48–51). A. fumigatus requires extracellular calcium to induce paradoxical growth upon caspofungin treatment (51). Here, we observed that adding extracellular calcium increased the MIC of caspofungin and fungal survival in both the wild type and mutants, confirming a direct connection between calcium levels and drug resistance in C. neoformans.

Calcineurin is an important regulator of Ca^2+^ signaling pathways (23). Strains with altered calcineurin function are known to be more susceptible to caspofungin (15, 22). *In vitro* studies demonstrated that the calcineurin inhibitor FK506 has a synergistic interaction with caspofungin against C. neoformans (24). In our study, we also found that the calcineurin inhibitor CsA increased caspofungin susceptibility in C. neoformans. In the presence of CsA, the MIC of caspofungin was much higher in the *cdc50*Δ mutant than in the *crm1*Δ *cdc50*Δ double mutant. We speculate that this occurs due to an inability of the double mutant to produce sufficient [Ca^2+^]c to resist caspofungin stress, while the *cdc50*Δ single mutant contains higher residual calcium levels. Consistent with this hypothesis, the caspofungin MIC for the double mutant increased dramatically when extracellular Ca^2+^ was added while the MIC for the *cdc50*Δ mutant showed only a 2-fold increase even with addition of 20 mM extracellular calcium (Table 1). In addition, increased gene expression in the calcineurin pathway in the presence of caspofungin in both wild-type and mutant strains supports the conclusion that caspofungin treatment activates the calcineurin signaling pathway in C. neoformans. Overall, we conclude that the calcineurin pathway contributes to Cdc50-mediated caspofungin resistance.

Interestingly, we also observed that the *crm1*Δ *cdc50*Δ double mutant exhibited higher CsA sensitivity than other strains (Table 2). We suspect this increased sensitivity may be related to an additional CsA function other than that of calcineurin inhibitor. For instance, FK506 has synergistic antifungal activity via a mechanism that is independent of calcineurin in C. neoformans (24). Calcineurin inhibitors FK506 and CsA were found to inhibit multidrug resistance pump functions in addition to calcineurin (52). Therefore, it is likely that deletion of both *CRM1* and *CDC50* leads to a synergistic effect that further sensitizes mutant cells against CsA. The exact mechanism remains unclear and requires future studies.

Calcineurin regulates intracellular calcium homeostasis in fungi (23). Calcium plays key roles in regulating cell death as intracellular Ca^2+^ overload triggers cell death (35). Stimulation of cells with calcium ionophores rapidly elevated [Ca^2+^]c, followed by a sequence of Ca^2+^-dependent signaling events, including externalization of PS, and apoptosis (53). Thus, we suspect that the excessive [Ca^2+^]c in the *cdc50*Δ mutant during caspofungin treatment may induce cell death and that increased concentrations of free cytoplasmic calcium may also trigger PS externalization (54). Indeed, caspofungin treatment significantly increased both PS exposure and ROS level in the *cdc50*Δ mutant compared to levels in other strains. Our results indicate that Crm1 is required for maintaining Ca^2+^ homeostasis under the caspofungin treatment, likely by supplying cytosolic free Ca^2+^ from ER and vacuole intracellular stores.

It has been shown that the calcineurin pathway was required for an increase in compensatory chitin content following echinocandin treatment (18–21, 55). Cell wall biosynthesis gene expression is altered during caspofungin treatment in C. neoformans (15). No significant decrease in chitin level occurred following caspofungin treatment in our study. Interestingly, *cdc50*Δ cells showed a higher level of chitosan than the wild type or the *crm1*Δ mutant, and the chitosan level of the *cdc50*Δ mutant decreased significantly following caspofungin treatment. Loss of *CRM1* in the *cdc50*Δ mutant rescued its reduced chitosan to maintain cell wall integrity. This process may partially explain why loss of Crm1 in the *cdc50*Δ mutant can rescue its caspofungin sensitivity.

Increased [Ca^2+^]c in human erythrocytes inhibits their ability to incorporate spin-labeled aminophospholipids, suggesting that [Ca^2+^]c may regulate flippase activity (56). ATP11C is one of the human P4-type ATPases localized to the plasma membrane in a Cdc50-dependent manner. High [Ca^2+^]c inhibits ATP11C flippase activity (57). Whether Ca^2+^ directly binds to the flippase remains an area for future investigation. In this study, we detected an interaction between Cdc50 and the mechanosensitive channel protein Crm1 and much higher [Ca^2+^]c in the *cdc50*Δ mutant. Based on our data, we conclude that Cdc50 is a likely regulator for both lipid flippase function and calcium channel regulation. How and when Cdc50 interacts with P4-ATPases versus Crm1, as well as the potential temporal and spatial regulation of these two biological processes, are topics for future investigation.

**FIG 8:**
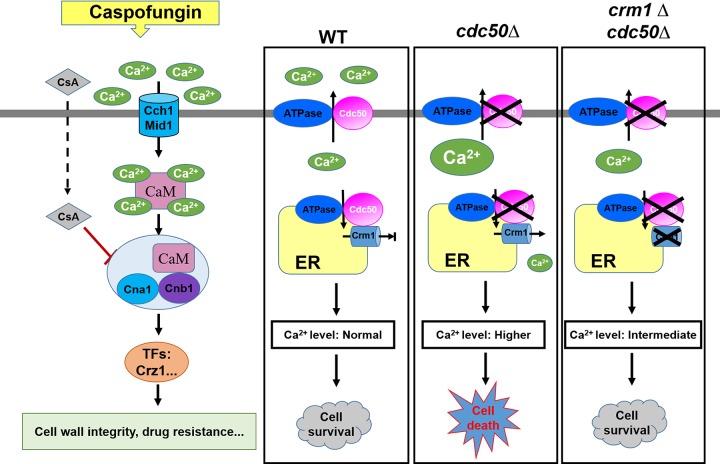
Model of the caspofungin resistance mechanism in C. neoformans. Caspofungin (CAS) treatment activates the Ca^2+^-calcineurin pathway, which regulates downstream targets that regulate cell wall integrity and drug resistance. In the wild type (WT), Cdc50 may coordinate Ca^2+^ efflux, which in conjunction with Crm1 plays roles in Ca^2+^ influx processes to maintain intracellular calcium homeostasis. An excessive elevation of intracellular calcium levels the in *cdc50*Δ mutant induces cell death. Loss of Crm1 in the *cdc50*Δ mutant alleviates the increased calcium levels. TF, transcription factor.

Based on our data, we propose a model depicting how Crm1 and Cdc50 contribute to regulating caspofungin resistance in C. neoformans (Fig. 8). With caspofungin treatment, Ca^2+^ enters the cell through high-affinity calcium channels, Cch1 and Mid1. This process activates the calcium-binding protein calmodulin (CaM), which binds to the calcineurin heterodimer (CnA and CnB) and enhances calcineurin phosphatase activity. The activated calcineurin complex dephosphorylates the target genes involved in regulating the stress response, cell wall integrity, growth, and drug resistance. In C. neoformans, calcium homeostasis can be maintained through the function of an elaborate system containing calcium channels and pumps. Crm1 plays a key role in Ca^2+^ elevation by transporting free Ca^2+^ from the ER to the cytosol. Cdc50 likely contributes to inhibiting the function of Crm1 to maintain intracellular calcium homeostasis. If the function of Cdc50 is impaired, uncontrolled Ca^2+^ influx by Crm1 leads to a lethal level of [Ca^2+^]c in the *cdc50*Δ mutant. Accordingly, loss of Crm1 in the *cdc50*Δ mutant alleviates the increased [Ca^2+^]c, and the cells become resistant to caspofungin.

In summary, our study on suppressor mutations of the *cdc50*Δ mutant in caspofungin sensitivity identified Crm1, a novel EF-MSL protein that plays an important role in regulating caspofungin resistance in C. neoformans by altering intracellular calcium homeostasis. The identification and characterization of Cdc50 and Crm1 in the calcium regulation of caspofungin resistance not only provide new insights for Cdc50-mediated echinocandin resistance in C. neoformans but also may advance our knowledge of calcium signaling in the fungal response to echinocandin drugs in general.

## MATERIALS AND METHODS

### Strains and medium.

Two C. neoformans gene deletion collections were generated by Hiten Madhani’s group at the University of California San Francisco (UCSF) and purchased from the American Tissue Culture Center (ATCC) and Fungal Genetics Stock Center (FGSC). Other strains used in this study are listed in Table 3. In all growth assays, cells were grown in nutrient-rich yeast extract-peptone-dextrose (YPD) medium at 30°C. The boron dipyrromethene difluoride (BODIPY)-labeled caspofungin was kindly provided by our colleague David Perlin. Caspofungin was provided by Merck. Other common medium preparation and growth conditions followed previously described instructions (58). Whole genome sequencing data were deposited to NCBI Sequence Read Archive (SRA) database with the accession no. PRJNA577954.

**TABLE 3:** Strains used in this study

C. neoformans strain	Genotype	Source or reference
H99	*MAT*α wild type	67
KN99**a**	*MAT***a** wild type	68
CUX196	*MAT*α *cdc50Δ*::*NEO*	31
CUX202	*MAT*α *cdc50Δ*::*NEO CDC50-GFP-NAT*	31
CUX759	*MAT*α *crm1 Δ*::*NEO*	69
CUX994	*MAT***a** *cdc50*Δ::*NEO*	This study
CUX995	*MAT*α *cdc50*Δ::*NEO crm1*Δ::*NAT*	This study
CUX1036	*MAT*α *cdc50Δ*::*NEO CDC50-GFP-NAT ura5*Δ	This study
CUX1037	*MAT*α *cdc50*Δ::*NEO crm1*Δ::*NAT ura5*Δ	This study
CUX1053	*MAT*α *cdc50*Δ::*NEO crm1*Δ::*NAT CRM1* (H99)::*URA5*	This study
CUX1054	*MAT*α *cdc50*Δ::*NEO crm*Δ::*NAT CRM1* (M1)::*URA5*	This study
CUX1063	*MAT*α *cdc50Δ*::*NEO CDC50-GFP-NAT ura5*Δ	This study
CUX1091	*MAT***a** *CRM1-mCherry-NAT*	This study
CUX1092	*MAT*α *CDC50-GFP-NAT*	This study
CUX1096	*MAT*α *CRM1-mCherry-NAT CDC50-GFP-NAT*	This study

### Generation of double mutants and *CRM1* complemented strain.

Genes with nonsynonymous mutations in both the M1 and M2 genomes were identified through genome comparison following whole-genome resequencing using the sequencing facility at Rutgers. Their mutants (mating type α) identified from the deletion collections were genetically crossed with a mating type **a** *cdc50*Δ mutant to generate double mutants. Cells from each culture were mixed and inoculated on Musahige-Skoog (MS) mating medium. Plates were incubated at room temperature in the dark for 10 days. Spores were dissected and inoculated on YPD plates containing both nourseothricin (NAT) and G418 and incubated at 30°C for 3 days. Genomic DNAs of progenies were extracted, and diagnostic PCR was performed to screen for double mutants.

To complement *CRM1* in the *crm1*Δ *cdc50*Δ double deletion strain, two PCR fragments containing a 1.5-kb upstream promoter region and the *CRM1* ORF and its 500 bp downstream region were amplified from H99 and M1 isolate genomic DNA using primers CX1499/CX1500 (see Table S1 in the supplemental material). The two PCR fragments were cloned into the plasmid pJAF7, which contains a *URA5* marker, by infusion cloning (TaKaRa). The linearized plasmids were biolistically transformed into the *crm1*Δ *cdc50*Δ double mutant to generate the complemented strains CUX1053 and CUX1054.

### Testing caspofungin sensitivity on agar medium.

YPD agar plates containing 8, 16, 32, or 64 μg/ml caspofungin were prepared. C. neoformans overnight cultures were collected, washed, and adjusted to a final concentration at the *A*_600_ of 1.0. Tenfold serial dilutions were prepared, and 5 μl of each sample was inoculated on agar plates. Plates were incubated for 4 days at 30°C before being photographed.

### Assays for melanin production and stress response.

Melanin production was assayed by inoculating C. neoformans cells into 2 ml of YPD liquid medium and incubating samples overnight at 30°C. Overnight cultures with serial dilutions were placed on l-3,4-dihydroxyphenylalanine (l-DOPA) agar medium. The agar plates were incubated at 30°C for 4 days before fungal colonies were assessed for pigmentation.

To assay for stress responses, 10-fold serial dilutions of yeast overnight cultures were prepared, and 5 μl each sample was spotted on YPD agar plates containing 1.0 M KCl for osmotic stress (59). To test cell wall integrity, cells were also spotted on YPD agar plates containing 0.03% SDS, 0.5% Congo red, or 250 μg/ml calcofluor white (CFW) and incubated for 4 days at 30°C or 37°C.

### *Cryptococcus-*macrophage interaction assay.

A *Cryptococcus*-macrophage interaction assay was performed as previously described (59). Macrophage-like cell line J774 cells were cultured in Dulbecco’s modified Eagle’s medium (DMEM) with 10% heat-inactivated fetal bovine serum (FBS) at 37°C with 5% CO_2_. J774 cells (5 × 10^4^) in 0.5 ml of fresh DMEM was added into each well of a 48-well culture plate, incubated at 37°C in 5% CO_2_ overnight, and activated with 50 unit/ml gamma interferon (IFN-γ; Invitrogen) and 1 μg/ml lipopolysaccharide (LPS; Sigma). C. neoformans overnight cultures were washed with phosphate-buffered saline (PBS) twice and opsonized with 20% mouse complement. *Cryptococcus* cells (2 × 10^5^) were added into each well (yeast/J774 ratio, 4:1). To assess the phagocytosis rate, cells were washed with PBS after a 2-h coincubation and fixed with methanol for 30 min. Giemsa stain was added to the wells at a 1:10 dilution, and the plates were incubated overnight at 4°C. Cells were washed once with PBS and analyzed using an inverted microscope. To assess intracellular proliferation of C. neoformans, nonadherent extracellular yeast cells were removed by gentle washing with fresh DMEM after a 2-h coincubation, and cultures were incubated for another 0, 4, and 24 h. At indicated time points on the figures, the medium in each well was replaced by distilled H_2_O (dH_2_O) to lyse macrophages for 30 min at room temperature. The lysate was spread on YPD plates, and the number of CFU/well was counted to determine intracellular proliferation.

### Quantitative RT-PCR.

C. neoformans cultures were grown overnight in YPD medium. Cells were washed twice in PBS, and 10^5^ cells/ml was replated onto YPD medium containing 0 or 8 μg/ml caspofungin. Cultures were incubated for 16 h at 30°C before cells were collected, and total RNA was extracted. The first-strand cDNA was synthesized according to a previously described protocol (60). Gene expression was analyzed using SYBR Advantage quantitative PCR (QPCR) premix reagents (TaKaRa). Gene expression levels were normalized using the endogenous housekeeping gene *GAPDH*, and the relative levels were determined using the comparative threshold cycle (*C_T_*) method (61). Real-time PCRs were performed using an Mx4000 QPCR system (Stratagene) as previously described (62).

### [Ca^2+^]c detection.

Cytosolic Ca^2+^ levels were measured by using a fluorescence-activated cell sorting (FACS) assay with Fluo-3 acetoxymethyl ester (Fluo-3/AM) indicator as previously described, with modifications (63). Briefly, C. neoformans cultures were grown overnight in YPD medium. Yeast cells (1 × 10^7^) were washed twice in d-Hanks buffer (0.4 g/liter KCl, 0.06 g/liter KH_2_PO_4_, 8.0 g/liter NaCl, 0.35 g/liter NaHCO_3_, 0.06 g/liter Na_2_HPO_4_·7H_2_O) and stained with 5 μM calcium-sensitive indicator Fluo-3/AM (Invitrogen) in the dark at 37°C for 30 min. Cells were then washed three times with d-Hanks buffer, resuspended into fresh liquid YPD medium containing 5 mM CaCl_2_ with either 0 or 4 μg/ml caspofungin, and incubated at 30°C. The concentration of intracellular free calcium ([Ca^2+^]c) at different time points was determined by flow cytometry using the excitation/emission wave lengths of 485 nm/530 nm. A 1-h time course and measurement at 16 h posttreatment were used to investigate the transient and long-term intracellular calcium changes with caspofungin treatment, respectively. This experiment was done in triplicate. The following equation was used to determine [Ca^2+^]c for samples from measured values of fluorescence signal: [Ca^2+^]c = *K_d_* × (*F* − *F*_min_)/(*F*_max_ − *F*), where *K_d_* is the dissociation constant, *F*_min_ is the fluorescence intensity of the indicator in the absence of calcium (YPD with 10 mM EGTA), *F*_max_ is the fluorescence of the calcium-saturated indicator (YPD with 20 mM CaCl_2_), and *F* is the fluorescence at intermediate calcium levels.

### BODIPY-caspofungin localization assay.

Fungal cultures were grown overnight in YPD medium and resuspended in YPD medium with 5 mM CaCl_2_ for an additional 5 h. Fresh cultures were washed with PBS and coincubated with 5 μM BODIPY fluorescently labeled caspofungin for 30 min. Cell suspensions were then observed under a fluorescence microscope for cellular localization of the fluorescent signal. Fluorescent signal intensity for 100,000 cells was also quantified by flow cytometry.

### Annexin V assay.

Annexin V staining was performed according to our previous publication (31). Briefly, *Cryptococcus* strains were grown overnight in YPD medium, recultured in YPD medium with 5 mM CaCl_2_ containing 0 or 4 μg/ml caspofungin, and incubated at 30°C for 48 h. Cells were harvested and washed in binding buffer (10 mM HEPES, pH 7.4, 140 mM NaCl, and 2.5 mM CaCl_2_) and resuspended in 1 ml of binding buffer containing 5 μl of FITC-conjugated annexin V (Life Technologies, Inc.). After being incubated for 1 h at 30°C with shaking, cells were fixed by 3.7% formaldehyde for 10 min at 37°C and then washed in PBS containing 1% formaldehyde before observation under fluorescence microscopy. Fluorescent signal intensity was also quantified by flow cytometry.

### ROS measurement.

Reactive oxygen species (ROS) detection was done as previously reported (64). Cells were grown overnight in YPD medium at 30°C. The following day, cells were diluted and allowed to grow until the *A*_600_ reached 0.5. Dichlorodihydrofluorescein diacetate (H_2_DCFDA) (Invitrogen) at a final concentration of 10 μM was added, and cells were incubated for an additional 2 h. Cells were then washed to remove excess dye, resuspended in YPD medium containing 5 mM CaCl_2_ and either 0 or 4 μg/ml caspofungin, and incubated at 30°C. Because cells were loaded with H_2_DCFDA prior to caspofungin treatment, the dye worked well when the duration of the treatment was short (5 h) (65). After a 5-h treatment, cells were then harvested, washed with PBS, and resuspended in 1 ml of PBS. Fluorescence signal was analyzed using an Accuri flow cytometer (BD Bioscience).

### Chitin and chitosan measurement.

*Cryptococcus* chitin and chitosan measurements were done as described by Baker et al. (43). Briefly, fungal cultures were grown for 20 h in YPD medium. Cells were inoculated into fresh liquid YPD medium containing 5 mM CaCl_2_ and either 0 or 4 μg/ml of caspofungin and incubated at 30°C for 72 h. Cells were divided, and dry weights were measured. One aliquot of pelleted cells was treated with sodium bicarbonate and acetic anhydride at room temperature for 20 min, followed by 5 min at 100°C. Both cell aliquots were subsequently extracted with KOH at 80°C for 90 min. Samples were collected and suspended in 0.2 ml of McIlvaine’s buffer (0.2 M Na_2_HPO_4_, 0.1 M citric acid, pH 6.0) containing 100 μg of chitinase from Trichoderma viride (C8241; Sigma) and incubated for 2 days at 37°C. For colorimetric determination of *N*-acetylglucosamine (GlcNAc), the Morgan-Elson method was adapted for microplate readers essentially as previously described (66). One hundred microliters of each sample was transferred to 96-well low-evaporation microliter plates, and absorbance at 585 nm was recorded. Standard curves were prepared from stocks of 0.2 to 2.0 mM GlcNAc (Sigma). The data shown are cumulated from three independent experiments. Statistical analysis was done by a two-tailed *t* test.

### Detection of Cdc50-Crm1 interaction.

A split ubiquitin system (Dualsystem Biotech, Switzerland) was utilized to investigate the interaction between Cdc50 and Crm1 as previously described (37, 38). *CDC50* full-length cDNA was cloned into the yeast expression vector pNCW (with Cub fused to the N terminus of Cdc50). *CRM1* full-length cDNA was cloned into the pDL2XN vector (with the mutated C-terminal half of ubiquitin NubG protein fused to the Crm1 N terminus). All cDNA sequences were confirmed by DNA sequencing. Cub and NubG fusion constructs were cotransformed into the host yeast strain NMY32. Two constructs, pAI-Alg5 and pDL2-Alg5, express a fusion of the endogenous ER protein Alg5 to the Nub portion of yeast ubiquitin. pAI-Alg5 contains a wild-type Nub that interacts with the Cub portion of the ubiquitin from the bait vector and serves as a positive control. pDL2-Alg5 contains a Nub portion bearing an isoleucine-to-glycine mutation that prevents nonspecific interaction with the Cub portion from the bait vector and serves as a negative control. The interaction was determined by the growth of yeast transformants on medium lacking histidine or adenine and also by measuring β-galactosidase activity.

To confirm the interaction between Cdc50 and Crm1, *CRM1* full-length genomic DNA was amplified and cloned into a vector containing the *Cryptococcus* actin promoter and an mCherry coding sequence to generate the plasmid pCXU338. The *CDC50* full-length genomic DNA was amplified and cloned into a vector containing the *Cryptococcus* histone H3 promoter and a GFP coding sequence to generate the plasmid pCXU350. These two plasmids were linearized and introduced into KN99 and H99 to generate strains CUX1091 (Crm1-mCherry) and CUX1092 (Cdc50-GFP) (Table 3), respectively. The strain expressing both Crm1-mCherry and Cdc50-GFP (CUX1096) was generated by crossing CUX1091 and CUX1092. To test the interaction between Crm1 and Cdc50 *in vivo*, proteins were purified from strains H99, CUX1091, CUX1092, and CUX1096 and analyzed by immunoblotting with anti-GFP and anti-mCherry antibodies. Proteins were pulled down by using anti-mCherry or anti-GFP antibodies and then analyzed by Western blotting.

## References

[1] Park BJ, Wannemuehler KA, Marston BJ, Govender N, Pappas PG, Chiller TA 2009 Estimation of the current global burden of cryptococcal meningitis among persons living with HIV/AIDS. AIDS 23:525–530. doi:10.1097/QAD.0b013e328322ffac. 19182676

[2] Pukkila-Worley R, Mylonakis E 2008 Epidemiology and management of cryptococcal meningitis: developments and challenges. Expert Opin Pharmacother 9:551–560. doi:10.1517/14656566.9.4.551. 18312157

[3] Perfect JR, Dismukes WE, Dromer F, Goldman DL, Graybill JR, Hamill RJ, Harrison TS, Larsen RA, Lortholary O, Nguyen MH, Pappas PG, Powderly WG, Singh N, Sobel JD, Sorrell TC 2010 Clinical practice guidelines for the management of cryptococcal disease: 2010 update by the infectious diseases society of America. Clin Infect Dis 50:291–322. doi:10.1086/649858. 20047480 PMC5826644

[4] Kartsonis NA, Nielsen J, Douglas CM 2003 Caspofungin: the first in a new class of antifungal agents. Drug Resist Updat 6:197–218. doi:10.1016/S1368-7646(03)00064-5. 12962685

[5] Denning DW 2003 Echinocandin antifungal drugs. Lancet 362:1142–1151. doi:10.1016/S0140-6736(03)14472-8. 14550704

[6] Perlin DS 2011 Current perspectives on echinocandin class drugs. Future Microbiol 6:441–457. doi:10.2217/fmb.11.19. 21526945 PMC3913534

[7] Maligie MA, Selitrennikoff CP 2005 *Cryptococcus neoformans* resistance to echinocandins: (1,3)β-glucan synthase activity is sensitive to echinocandins. Antimicrob Agents Chemother 49:2851–2856. doi:10.1128/AAC.49.7.2851-2856.2005. 15980360 PMC1168702

[8] Perlin DS 2015 Mechanisms of echinocandin antifungal drug resistance. Ann N Y Acad Sci 1354:1–11. doi:10.1111/nyas.12831. 26190298 PMC4626328

[9] Gardiner RE, Souteropoulos P, Park S, Perlin DS 2005 Characterization of *Aspergillus fumigatus* mutants with reduced susceptibility to caspofungin. Med Mycol 43(Suppl 1):S299–S305. doi:10.1080/13693780400029023. 16110824

[10] Rocha EM, Garcia-Effron G, Park S, Perlin DS 2007 A Ser678Pro substitution in Fks1p confers resistance to echinocandin drugs in *Aspergillus fumigatus*. Antimicrob Agents Chemother 51:4174–4176. doi:10.1128/AAC.00917-07. 17724146 PMC2151465

[11] Walker LA, Gow NA, Munro CA 2010 Fungal echinocandin resistance. Fungal Genet Biol 47:117–126. doi:10.1016/j.fgb.2009.09.003. 19770064 PMC2812698

[12] Beyda ND, Lewis RE, Garey KW 2012 Echinocandin resistance in *Candida* species: mechanisms of reduced susceptibility and therapeutic approaches. Ann Pharmacother 46:1086–1096. doi:10.1345/aph.1R020. 22811350

[13] Shapiro RS, Robbins N, Cowen LE 2011 Regulatory circuitry governing fungal development, drug resistance, and disease. Microbiol Mol Biol Rev 75:213–267. doi:10.1128/MMBR.00045-10. 21646428 PMC3122626

[14] Imtiaz T, Lee KK, Munro CA, MacCallum DM, Shankland GS, Johnson EM, MacGregor MS, Bal AM 2012 Echinocandin resistance due to simultaneous *FKS* mutation and increased cell wall chitin in a *Candida albicans* bloodstream isolate following brief exposure to caspofungin. J Med Microbiol 61:1330–1334. doi:10.1099/jmm.0.045047-0. 22653922

[15] Pianalto KM, Billmyre RB, Telzrow CL, Alspaugh JA 2019 Roles for stress response and cell wall biosynthesis pathways in caspofungin tolerance in *Cryptococcus neoformans*. Genetics 213:213–227. doi:10.1534/genetics.119.302290. 31266771 PMC6727808

[16] Walker LA, Munro CA, De Bruijn I, Lenardon MD, McKinnon A, Gow N 2008 Stimulation of chitin synthesis rescues *Candida albicans* from echinocandins. PLoS Pathog 4:e1000040. doi:10.1371/journal.ppat.1000040. 18389063 PMC2271054

[17] Cota JM, Grabinski JL, Talbert RL, Burgess DS, Rogers PD, Edlind TD, Wiederhold NP 2008 Increases in *SLT2* expression and chitin content are associated with incomplete killing of *Candida glabrata* by caspofungin. Antimicrob Agents Chemother 52:1144–1146. doi:10.1128/AAC.01542-07. 18086838 PMC2258485

[18] Fortwendel JR, Juvvadi PR, Pinchai N, Perfect BZ, Alspaugh JA, Perfect JR, Steinbach WJ 2009 Differential effects of inhibiting chitin and 1,3-β-d-glucan synthesis in ras and calcineurin mutants of *Aspergillus fumigatus*. Antimicrob Agents Chemother 53:476–482. doi:10.1128/AAC.01154-08. 19015336 PMC2630655

[19] Walker LA, Gow NA, Munro CA 2013 Elevated chitin content reduces the susceptibility of *Candida* species to caspofungin. Antimicrob Agents Chemother 57:146–154. doi:10.1128/AAC.01486-12. 23089748 PMC3535899

[20] Fortwendel JR, Juvvadi PR, Perfect BZ, Rogg LE, Perfect JR, Steinbach WJ 2010 Transcriptional regulation of chitin synthases by calcineurin controls paradoxical growth of *Aspergillus fumigatus* in response to caspofungin. Antimicrob Agents Chemother 54:1555–1563. doi:10.1128/AAC.00854-09. 20124000 PMC2849361

[21] Munro CA, Selvaggini S, de Bruijn I, Walker L, Lenardon MD, Gerssen B, Milne S, Brown AJ, Gow NA 2007 The PKC, HOG and Ca^2+^ signalling pathways co-ordinately regulate chitin synthesis in *Candida albicans*. Mol Microbiol 63:1399–1413. doi:10.1111/j.1365-2958.2007.05588.x. 17302816 PMC2649417

[22] Kraus PR, Fox DS, Cox GM, Heitman J 2003 The *Cryptococcus neoformans* MAP kinase Mpk1 regulates cell integrity in response to antifungal drugs and loss of calcineurin function. Mol Microbiol 48:1377–1387. doi:10.1046/j.1365-2958.2003.03508.x. 12787363 PMC1635492

[23] Juvvadi PR, Lamoth F, Steinbach WJ 2014 Calcineurin as a multifunctional regulator: unraveling novel functions in fungal stress responses, hyphal growth, drug resistance, and pathogenesis. Fungal Biol Rev 28:56–69. doi:10.1016/j.fbr.2014.02.004. 25383089 PMC4219591

[24] Del Poeta M, Cruz MC, Cardenas ME, Perfect JR, Heitman J 2000 Synergistic antifungal activities of bafilomycin A_1_, fluconazole, and the pneumocandin MK-0991/caspofungin acetate (L-743,873) with calcineurin inhibitors FK506 and L-685,818 against *Cryptococcus neoformans*. Antimicrob Agents Chemother 44:739–746. doi:10.1128/aac.44.3.739-746.2000. 10681348 PMC89756

[25] Onyewu C, Afshari NA, Heitman J 2006 Calcineurin promotes infection of the cornea by *Candida albicans* and can be targeted to enhance fluconazole therapy. Antimicrob Agents Chemother 50:3963–3965. doi:10.1128/AAC.00393-06. 16923949 PMC1635197

[26] Uppuluri P, Nett J, Heitman J, Andes D 2008 Synergistic effect of calcineurin inhibitors and fluconazole against *Candida albicans* biofilms. Antimicrob Agents Chemother 52:1127–1132. doi:10.1128/AAC.01397-07. 18180354 PMC2258509

[27] Yang F, Zhang L, Wakabayashi H, Myers J, Jiang Y, Cao Y, Jimenez-Ortigosa C, Perlin DS, Rustchenko E 2017 Tolerance to caspofungin in *Candida albicans* is associated with at least three distinctive mechanisms that govern expression of *FKS* genes and cell wall remodeling. Antimicrob Agents Chemother 61:e00071-17. doi:10.1128/AAC.00071-17. 28223384 PMC5404545

[28] Chen YL, Yu SJ, Huang HY, Chang YL, Lehman VN, Silao FG, Bigol UG, Bungay AA, Averette A, Heitman J 2014 Calcineurin controls hyphal growth, virulence, and drug tolerance of *Candida tropicalis*. Eukaryot Cell 13:844–854. doi:10.1128/EC.00302-13. 24442892 PMC4135728

[29] Juvvadi PR, Lee SC, Heitman J, Steinbach WJ 2017 Calcineurin in fungal virulence and drug resistance: prospects for harnessing targeted inhibition of calcineurin for an antifungal therapeutic approach. Virulence 8:186–197. doi:10.1080/21505594.2016.1201250. 27325145 PMC5354160

[30] Lev S, Desmarini D, Chayakulkeeree M, Sorrell TC, Djordjevic JT 2012 The Crz1/Sp1 transcription factor of *Cryptococcus neoformans* is activated by calcineurin and regulates cell wall integrity. PLoS One 7:e51403. doi:10.1371/journal.pone.0051403. 23251520 PMC3520850

[31] Huang W, Liao G, Baker GM, Wang Y, Lau R, Paderu P, Perlin DS, Xue C 2016 Lipid flippase subunit Cdc50 mediates drug resistance and virulence in *Cryptococcus neoformans*. mBio 7:e00478-16. 27165800 10.1128/mBio.00478-16 PMC4959666

[32] Lenoir G, Williamson P, Puts CF, Holthuis JC 2009 Cdc50p plays a vital role in the ATPase reaction cycle of the putative aminophospholipid transporter Drs2p. J Biol Chem 284:17956–17967. doi:10.1074/jbc.M109.013722. 19411703 PMC2709398

[33] Thompson JR, Douglas CM, Li WL, Jue CK, Pramanik B, Yuan XL, Rude TH, Toffaletti DL, Perfect JR, Kurtz M 1999 A glucan synthase *FKS1* homolog in *Cryptococcus neoformans* is single copy and encodes an essential function. J Bacteriol 181:444–453. 9882657 10.1128/jb.181.2.444-453.1999 PMC93397

[34] Haswell ES, Meyerowitz EM 2006 MscS-like proteins control plastid size and shape in *Arabidopsis thaliana*. Curr Biol 16:1–11. doi:10.1016/j.cub.2005.11.044. 16401419

[35] Orrenius S, Zhivotovsky B, Nicotera P 2003 Regulation of cell death: the calcium-apoptosis link. Nat Rev Mol Cell Biol 4:552–565. doi:10.1038/nrm1150. 12838338

[36] Eisenberg T, Carmona-Gutierrez D, Buttner S, Tavernarakis N, Madeo F 2010 Necrosis in yeast. Apoptosis 15:257–268. doi:10.1007/s10495-009-0453-4. 20238475

[37] Xue C, Bahn YS, Cox GM, Heitman J 2006 G protein-coupled receptor Gpr4 senses amino acids and activates the cAMP-PKA pathway in *Cryptococcus neoformans*. Mol Biol Cell 17:667–679. doi:10.1091/mbc.e05-07-0699. 16291861 PMC1356578

[38] Hsueh YP, Xue C, Heitman J 2007 G protein signaling governing cell fate decisions involves opposing Gα subunits in *Cryptococcus neoformans*. Mol Biol Cell 18:3237–3249. doi:10.1091/mbc.e07-02-0133. 17581859 PMC1951760

[39] Pratt A, Garcia-Effron G, Zhao YN, Park S, Mustaev A, Pillai S, Perlin DS 2013 Evaluation of fungal-specific fluorescent labeled echinocandin probes as diagnostic adjuncts. Med Mycol 51:103–107. doi:10.3109/13693786.2012.685767. 22587729

[40] Araki Y, Ito E 1974 A pathway of chitosan formation in *Mucor rouxii*: enzymatic deacetylation of chitin. Biochem Biophys Res Commun 56:669–675. doi:10.1016/0006-291x(74)90657-3. 4826874

[41] Kafetzopoulos D, Martinou A, Bouriotis V 1993 Bioconversion of chitin to chitosan: purification and characterization of chitin deacetylase from *Mucor rouxii*. Proc Natl Acad Sci U S A 90:2564–2568. doi:10.1073/pnas.90.7.2564. 8464862 PMC46135

[42] Camacho E, Chrissian C, Cordero RJB, Liporagi-Lopes L, Stark RE, Casadevall A 2017 *N*-Acetylglucosamine affects *Cryptococcus neoformans* cell-wall composition and melanin architecture. Microbiology 163:1540–1556. doi:10.1099/mic.0.000552. 29043954 PMC5775898

[43] Baker LG, Specht CA, Donlin MJ, Lodge JK 2007 Chitosan, the deacetylated form of chitin, is necessary for cell wall integrity in *Cryptococcus neoformans*. Eukaryot Cell 6:855–867. doi:10.1128/EC.00399-06. 17400891 PMC1899242

[44] Nakayama Y, Iida H 2014 Organellar mechanosensitive channels involved in hypo-osmoregulation in fission yeast. Cell Calcium 56:467–471. doi:10.1016/j.ceca.2014.10.001. 25454595

[45] Malcolm HR, Maurer JA 2012 The mechanosensitive channel of small conductance (MscS) superfamily: not just mechanosensitive channels anymore. Chembiochem 13:2037–2043. doi:10.1002/cbic.201200410. 22915507

[46] Nakayama Y, Yoshimura K, Iida H 2012 Organellar mechanosensitive channels in fission yeast regulate the hypo-osmotic shock response. Nat Commun 3:1020. doi:10.1038/ncomms2014. 22910366

[47] Nakayama Y, Hirata A, Iida H 2014 Mechanosensitive channels Msy1 and Msy2 are required for maintaining organelle integrity upon hypoosmotic shock in *Schizosaccharomyces pombe*. FEMS Yeast Res 14:992–994. doi:10.1111/1567-1364.12181. 25041276

[48] Stevens DA, Espiritu M, Parmar R 2004 Paradoxical effect of caspofungin: reduced activity against *Candida albicans* at high drug concentrations. Antimicrob Agents Chemother 48:3407–3411. doi:10.1128/AAC.48.9.3407-3411.2004. 15328104 PMC514730

[49] Chamilos G, Lewis RE, Albert N, Kontoyiannis DP 2007 Paradoxical effect of echinocandins across *Candida* species in vitro: evidence for echinocandin-specific and candida species-related differences. Antimicrob Agents Chemother 51:2257–2259. doi:10.1128/AAC.00095-07. 17438060 PMC1891358

[50] Antachopoulos C, Meletiadis J, Sein T, Roilides E, Walsh TJ 2007 Concentration-dependent effects of caspofungin on the metabolic activity of *Aspergillus* species. Antimicrob Agents Chemother 51:881–887. doi:10.1128/AAC.01160-06. 17145783 PMC1803126

[51] Juvvadi PR, Munoz A, Lamoth F, Soderblom EJ, Moseley MA, Read ND, Steinbach WJ 2015 Calcium-mediated induction of paradoxical growth following caspofungin treatment is associated with calcineurin activation and phosphorylation in *Aspergillus fumigatus*. Antimicrob Agents Chemother 59:4946–4955. doi:10.1128/AAC.00263-15. 26055379 PMC4505252

[52] Mizuno K, Furuhashi Y, Misawa T, Iwata M, Kawai M, Kikkawa F, Kano T, Tomoda Y 1992 Modulation of multidrug resistance by immunosuppressive agents: cyclosporin analogues, FK506 and mizoribine. Anticancer Res 12:21–25. 1373592

[53] Parekh AB, Putney JW Jr. 2005 Store-operated calcium channels. Physiol Rev 85:757–810. doi:10.1152/physrev.00057.2003. 15788710

[54] Bevers EM, Comfurius P, Dekkers DW, Zwaal RF 1999 Lipid translocation across the plasma membrane of mammalian cells. Biochim Biophys Acta 1439:317–330. doi:10.1016/s1388-1981(99)00110-9. 10446420

[55] Juvvadi PR, Fortwendel JR, Rogg LE, Burns KA, Randell SH, Steinbach WJ 2011 Localization and activity of the calcineurin catalytic and regulatory subunit complex at the septum is essential for hyphal elongation and proper septation in *Aspergillus fumigatus*. Mol Microbiol 82:1235–1259. doi:10.1111/j.1365-2958.2011.07886.x. 22066998 PMC3225650

[56] Bitbol M, Fellmann P, Zachowski A, Devaux PF 1987 Ion regulation of phosphatidylserine and phosphatidylethanolamine outside-inside translocation in human erythrocytes. Biochim Biophys Acta 904:268–282. doi:10.1016/0005-2736(87)90376-2. 3117114

[57] Segawa K, Kurata S, Nagata S 2016 Human type IV P-type ATPases that work as plasma membrane phospholipid flippases and their regulation by caspase and calcium. J Biol Chem 291:762–772. doi:10.1074/jbc.M115.690727. 26567335 PMC4705396

[58] Wang YN, Liu TB, Delmas G, Park S, Perlin D, Xue CY 2011 Two major inositol transporters and their role in cryptococcal virulence. Eukaryot Cell 10:618–628. doi:10.1128/EC.00327-10. 21398509 PMC3127654

[59] Liu TB, Xue C 2014 Fbp1-mediated ubiquitin-proteasome pathway controls *Cryptococcus neoformans* virulence by regulating fungal intracellular growth in macrophages. Infect Immun 82:557–568. doi:10.1128/IAI.00994-13. 24478071 PMC3911387

[60] Liu TB, Wang Y, Stukes S, Chen Q, Casadevall A, Xue C 2011 The F-box protein Fbp1 regulates sexual reproduction and virulence in *Cryptococcus neoformans*. Eukaryot Cell 10:791–802. doi:10.1128/EC.00004-11. 21478432 PMC3127668

[61] Livak KJ, Schmittgen TD 2001 Analysis of relative gene expression data using real-time quantitative PCR and the 2^−ΔΔCT^ method. Methods 25:402–408. doi:10.1006/meth.2001.1262. 11846609

[62] Xue C, Liu T, Chen L, Li W, Liu I, Kronstad JW, Seyfang A, Heitman J 2010 Role of an expanded inositol transporter repertoire in *Cryptococcus neoformans* sexual reproduction and virulence. mBio 1:e00084-10. doi:10.1128/mBio.00084-10. 20689743 PMC2912663

[63] Shi W, Chen Z, Chen X, Cao L, Liu P, Sun S 2010 The combination of minocycline and fluconazole causes synergistic growth inhibition against *Candida albicans*: an in vitro interaction of antifungal and antibacterial agents. FEMS Yeast Res 10:885–893. doi:10.1111/j.1567-1364.2010.00664.x. 20707818

[64] Ingavale SS, Chang YC, Lee H, McClelland CM, Leong ML, Kwon-Chung KJ 2008 Importance of mitochondria in survival of *Cryptococcus neoformans* under low oxygen conditions and tolerance to cobalt chloride. PLoS Pathog 4:e1000155. doi:10.1371/journal.ppat.1000155. 18802457 PMC2528940

[65] Wu D, Yotnda P 2011 Production and detection of reactive oxygen species (ROS) in cancers. J Vis Exp 57:e357. doi:10.3791/3357. PMC3308605 22127014

[66] Masso-Silva J, Espinosa V, Liu TB, Wang Y, Xue C, Rivera A 2018 The F-box protein Fbp1 shapes the immunogenic potential of *Cryptococcus neoformans*. mBio 9:e01828-17. doi:10.1128/mBio.01828-17. 29317510 PMC5760740

[67] Perfect JR, Schell WA, Rinaldi MG 1993 Uncommon invasive fungal pathogens in the acquired immunodeficiency syndrome. J Med Vet Mycol 31:175–179. doi:10.1080/02681219380000211. 8509954

[68] Nielsen K, Cox GM, Wang P, Toffaletti DL, Perfect JR, Heitman J 2003 Sexual cycle of *Cryptococcus neoformans* var. *grubii* and virulence of congenic a and α isolates. Infect Immun 71:4831–4841. doi:10.1128/iai.71.9.4831-4841.2003. 12933823 PMC187335

[69] Jung KW, Yang DH, Maeng S, Lee KT, So YS, Hong J, Choi J, Byun HJ, Kim H, Bang S, Song MH, Lee JW, Kim MS, Kim SY, Ji JH, Park G, Kwon H, Cha S, Meyers GL, Wang LL, Jang J, Janbon G, Adedoyin G, Kim T, Averette AK, Heitman J, Cheong E, Lee YH, Lee YW, Bahn YS 2015 Systematic functional profiling of transcription factor networks in *Cryptococcus neoformans*. Nat Commun 6:6757. doi:10.1038/ncomms7757. 25849373 PMC4391232

