# Supplementary figures and images for: A Mechanosensitive Channel Governs Lipid Flippase-Mediated Echinocandin Resistance in Cryptococcus neoformans
Source: mBio. 2019 Dec 10;10(6):e01952-19. doi: 10.1128/mBio.01952-19 (PMC6904872; doi:10.1128/mBio.01952-19)

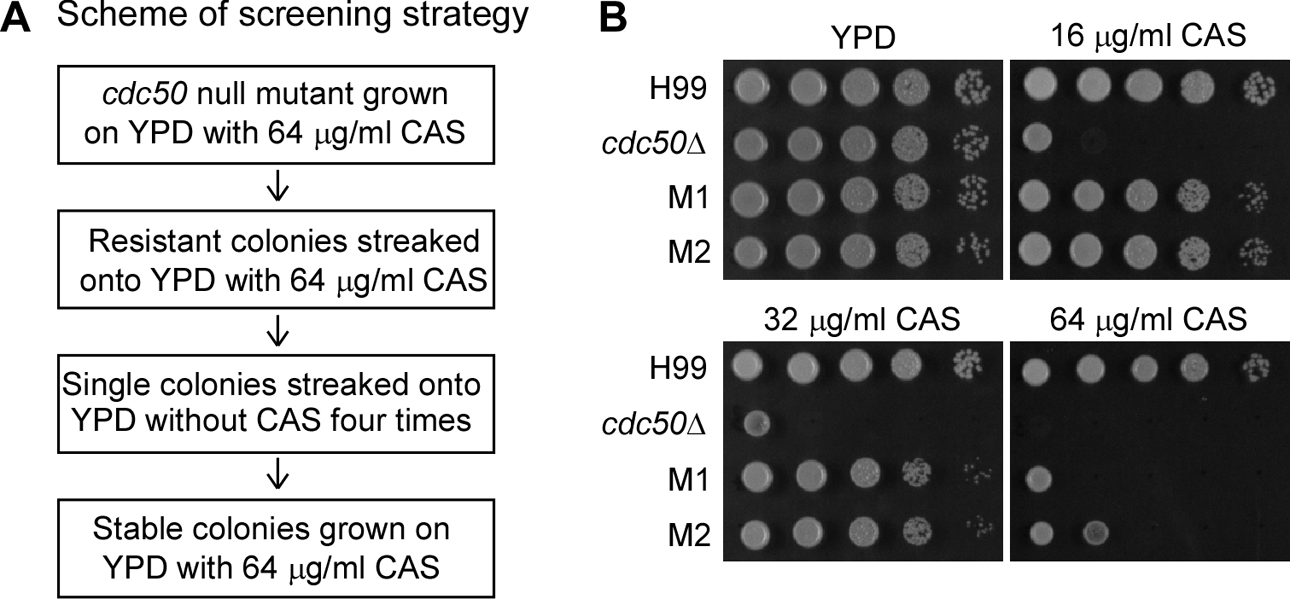

Supplement: FIG S1 [file mBio.01952-19-sf001.tif]

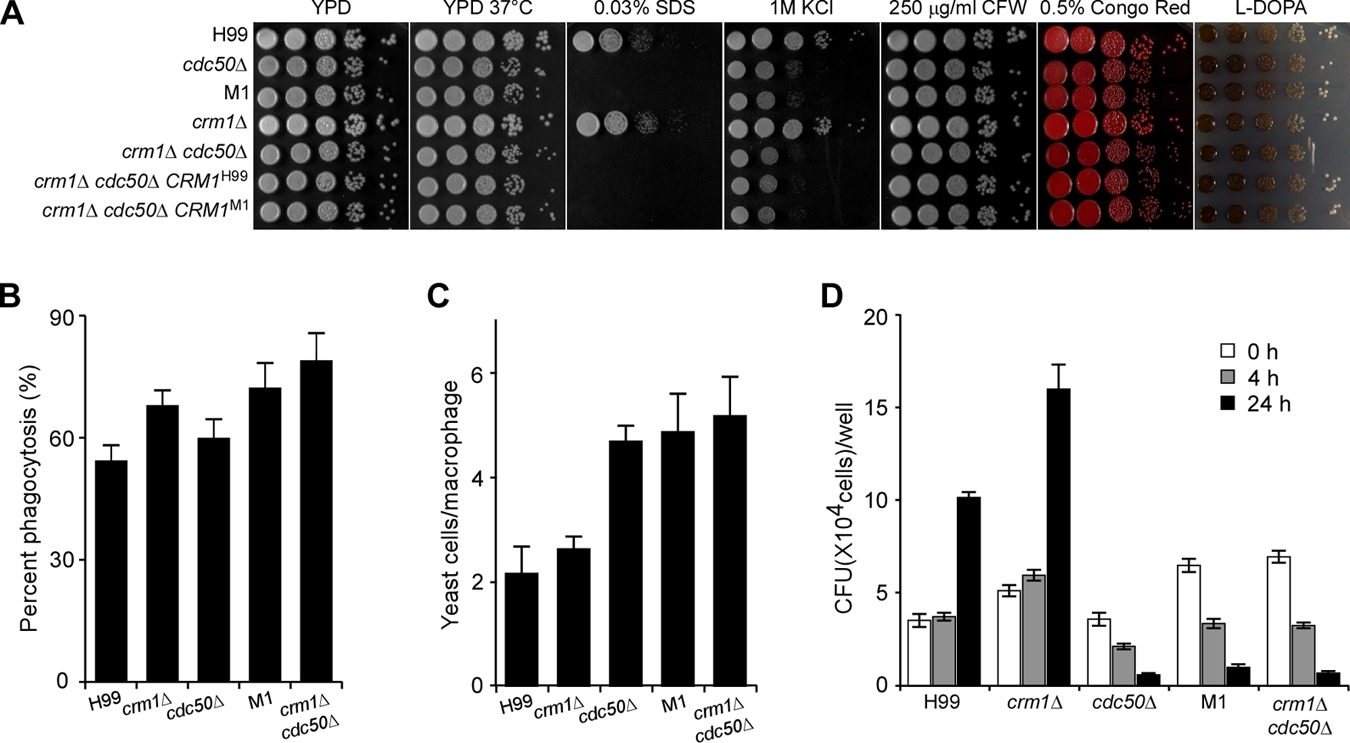

Supplement: FIG S2 [file mBio.01952-19-sf002.tif]
