# Supplementary material for: A Mechanosensitive Channel Governs Lipid Flippase-Mediated Echinocandin Resistance in Cryptococcus neoformans
Source: mBio. 2019 Dec 10;10(6):e01952-19. doi: 10.1128/mBio.01952-19 (PMC6904872; doi:10.1128/mBio.01952-19)
Supplement: TABLE S1 [file mBio.01952-19-st001.docx]

Table S1. Genes contain mutations in M1 and M2

| **Gene number** | **M1** | **M2** | **M1 & M2** |
| --- | --- | --- | --- |
| CNAG_00014 | I298V Q301R |  | No |
| CNAG_00483 | E4I E6Q | E4I E6Q | Yes |
| CNAG_01704 | P115fs Q114K | Q377P | Yes |
| CNAG_02989 |  | L225fs | No |
| CNAG_03507 | S20P G18A S11P S10R | S20P G18A | Yes |
| CNAG_05436 | A251T V293M  A298V R321C | V293M A298V | Yes |
| CNAG_07653 | H10R |  | No |
| CNAG_07704 | R81* I91L  I109T S111G  V113I H114R  G115A | R81* I91L  I109T S111G  V113I H114R  G115A | Yes |

fs: nucleotides insertion or deletion

*: Stop codon
