# Supplementary material for: A Mechanosensitive Channel Governs Lipid Flippase-Mediated Echinocandin Resistance in Cryptococcus neoformans
Source: mBio. 2019 Dec 10;10(6):e01952-19. doi: 10.1128/mBio.01952-19 (PMC6904872; doi:10.1128/mBio.01952-19)
Supplement: TABLE S2 [file mBio.01952-19-st002.docx]

Table S2. MICs of caspofungin in YPD supplemented with Calcium chelator BAPTA

| Strains | BAPTA levels in YPD (µg/mL) | | | | | |
| --- | --- | --- | --- | --- | --- | --- |
|  | 0 | 8 | 16 | 32 | 64 | 128 |
| H99 | 16 | 16 | 16 | 8 | 8 | 8 |
| *cdc50*Δ | 4 | 4 | 4 | 4 | 4 | 2 |
| *crm1*Δ | 8 | 8 | 8 | 8 | 8 | 4 |
| *crm1*Δ *cdc50*Δ | 4 | 4 | 4 | 4 | 4 | 2 |
| *CRM1*^OE^ | 16 | 16 | 16 | 16 | 16 | 16 |
