# Supplementary material for: A Mechanosensitive Channel Governs Lipid Flippase-Mediated Echinocandin Resistance in Cryptococcus neoformans
Source: mBio. 2019 Dec 10;10(6):e01952-19. doi: 10.1128/mBio.01952-19 (PMC6904872; doi:10.1128/mBio.01952-19)
Supplement: TABLE S3 [file mBio.01952-19-st003.docx]

Table S3 Primers used in this study

| Primers | Sequences (5’-3’) | References/Note |
| --- | --- | --- |
| CX618 | CCGCTAGGTGTGAGATTGAGTTTG | *CDC50* F3 |
| CX619 | GAATCCGTCCACATAGCCGTTAGG | *CDC50* R3 |
| CX1116 | GCAAGAGCAGGAAGTATCTTG | *FKS1* F2 |
| CX1117 | GTGCTCGATCGAGAGAAGGTG | *FKS1* R2 |
| CX1118 | GCTTCCGATCGATAGATTCCT | *FKS1* F3 |
| CX1119 | GACCGACGACAGTAATCCAG | *FKS1* R3 |
| CX1425 | TCTGGTGTCTGGCAATCTG | *MSY1* F3/seqF2 |
| CX1426 | TTGGATGTCAAGATGAGCAGC | *MSY1* R3 |
| CX1488 | TCGTCACTTCCATCTCTTCC | *MSY1* seqF1 |
| CX1489 | ACGCAATATACGATCGAGCAG | *MSY1* seqR1 |
| CX1490 | ACTTCGAAAGCAAATTCTTC | *MSY1* seqR2 |
| CX1491 | ATGCAGCTCTTGTCGTCATC | *MSY1* seqF3 |
| CX1492 | ATCAGCCATTATCCGACCTC | *MSY1* seqR3 |
| CX1499 | AATTGCGGCCGCACGCTTTCATATGGCTTTTC | *MSY1* comp F |
| CX1500 | TATACTGCAGTCTAAGGCTGTCTCTTAGC | *MSY1* comp R |
| CX1526 | CAACATGTCTGGATCCATGTCAGACGATAGCGCGAC | *MSY1* OE F |
| CX1574 | TCTAGAACTAGTGGATCCAGGCTGTCTCTTAGCATCG | *MSY1* OE R |
| CX1578 | GCTGAGATTCGCTTTCAAGG | *CNB1* QPCR F |
| CX1579 | AGAGCTTTCCATCCCCATCT | *CNB1* QPCR R |
| CX1580 | TGTGGTCGCTCAAGATGTG | *CNA1* QPCR F |
| CX1581 | ACTTTCCATGCAGGCATTG | *CNA1* QPCR R |
| CX1582 | ACTCTGAGCCACTGAACTC | *MID1* QPCR F |
| CX1583 | GTCACCAACAGTCATCCA | *MID1* QPCR R |
| CX1584 | TCCGAACTTGTCCTTTACG | *CHS3* QPCR F |
| CX1585 | GGTCTGTAGATATCGTCCGT | *CHS3* QPCR R |
| CX1588 | AGCAGCACGAACGATAATC | *CCH1* QPCR F |
| CX1589 | ACAGTATGACAGGCTCCGTC | *CCH1* QPCR R |
